# Statement of Retraction: Circular RNA SOX13 promotes malignant behavior and cisplatin resistance in non-small cell lung cancer through targeting microRNA-3194-3p/microtubule-associated protein RP/EB family member 1

**DOI:** 10.1080/21655979.2024.2299528

**Published:** 2024-02-20

We, the Editors and Publisher of the journal Bioengineered, have retracted the following article:

Libin Zhang, Hao Peng, Zheyuan Xu, Qiuju Yang, Yang Wang, Han Wang & Liang Bu (2022) Circular RNA SOX13 promotes malignant behavior and cisplatin resistance in non-small cell lung cancer through targeting microRNA-3194-3p/microtubule-associated protein RP/EB family member 1, Bioengineered, 13:1, 1814-1827, DOI: 10.1080/21655979.2021.1997223

Following publication, the authors notified the journal that the results reported within the article may not be reproduceable. Following an investigation by the Publisher, significant additional concerns were identified regarding the compliance with the journal editorial policies and the integrity of the data. As the Editor and Publisher also have concerns about the integrity of the reported results, all parties have agreed to retract the article to ensure correction of the scholarly record.

We have been informed in our decision-making by our editorial policies and the COPE guidelines.

The retracted article will remain online to maintain the scholarly record, but it will be digitally watermarked on each page as ‘Retracted’.

